# Inhibition of the mammalian target of rapamycin leads to autophagy activation and cell death of MG63 osteosarcoma cells

**DOI:** 10.3892/ol.2013.1531

**Published:** 2013-08-16

**Authors:** ZONG-GANG XIE, YE XIE, QI-RONG DONG

**Affiliations:** Department of Orthopedic Surgery, The Second Affiliated Hospital, Soochow University, Suzhou, Jiangsu 215006, P.R. China

**Keywords:** mammalian target of rapamycin, rapamycin, autophagy, chemotherapy

## Abstract

It has been well documented that the inhibition of the mammalian target of rapamycin (mTOR) induces autophagy in proliferative cells. Therefore, mTOR inhibitors have been proposed for the treatment of cancer. As autophagy plays significant roles in tumor cell survival, the present study aimed to investigate the contribution of autophagy activation to the antitumor effects of cis-diamminedichloroplatinum (CDDP). An MTT assay was used to determine the cytotoxic effects of rapamycin on MG63 osteosarcoma cells. The cell cycle was assessed using a flow cytometry analysis subsequent to staining the DNA with propidium iodide. The mitochondrial membrane potential (Δψ) was measured using the fluorescent probe, JC-1. Western blot analysis was used to determine the expression of the proteins that are involved in apoptosis and autophagy, including p53, p62, light chain 3 (LC3) and Beclin-1. The viability of the MG63 cells was inhibited following rapamycin or CDDP treatment. The mitochondrial Δψ collapsed following treatment with rapamycin or CDDP. Rapamycin induced cell death and enhanced the effects of the induction of MG63 cell death by CDDP. Western blot analysis detected the induced expression of the p53 and Beclin-1 proteins and the autophagic proteins, LC3 and p62. Rapamycin was observed to induce the death of cancer cells through apoptotic and autophagic mechanisms. Rapamycin may enhance the effects of the activation of autophagy and the induction of apoptosis by CDDP.

## Introduction

Osteosarcoma is the most commonly diagnosed primary malignant tumor of the bone (1,2). The contemporary treatment of osteosarcoma requires multidisciplinary therapy, incorporating surgery and systemic chemotherapy (1,3). The prognosis of osteosarcoma patients has significantly improved with the advent of chemotherapy. In the pre-chemotherapy era, when patients underwent surgery as the only form of treatment, the survival rate was <20% (4). Osteoclasts have drawn attention as a therapeutic target in various bone disorders, including osteosarcoma. The osteoclast is the only cell that resorbs bone and is central to pathological situations, where bone destruction is intricately involved. Osteosarcoma cells are of the osteoblastic lineage. Hence, osteosarcoma is a more ideal candidate for osteoclast-targeted therapy than other primary and metastatic bone tumors. The rapid progress that has been made in understanding the molecular mechanism that regulates osteoclasts has propelled the development of new therapeutic approaches (5).

Autophagy is intricately implicated in health and disease. Autophagy defects play a role in the pathogenesis of numerous diseases, including myopathy, neuronal degeneration, microbial infection, inflammatory bowel disease, aging and cancer (6–11). Studies have demonstrated the functional role of autophagy in various cellular processes and the potential of autophagy modulation as a novel therapeutic strategy for a number of pathological conditions, including cancer (12–14).

Anticancer therapies, including hormonal agents, chemotherapy and irradiation, frequently induce autophagy, in most cases as a prosurvival response potentially contributing to treatment resistance. However, autophagy activation in particular genetic backgrounds and/or the completion of the autophagic process beyond the reversibility of cell viability may also lead to cell death, thus enhancing the efficacy of the treatment (15–18).

The activation of autophagy by the inhibition of the mammalian target of rapamycin (mTOR) may contribute to anti-tumor actions. The present study examined the effects of the mTOR inhibitor, rapamycin, on the activation of autophagy and the contribution of autophagy to the chemosensitivity effects of cis-diamminedichloroplatinum (CDDP) on osteosarcoma MG63 cells.

## Materials and methods

### Reagents

MG63 osteosarcoma cancer cells were purchased from the Shanghai Institute of Cell Biology, Chinese Academy of Sciences (Shanghai, China). RPMI-1640 medium was purchased from Gibco (Rockville, MD, USA) and rapamycin were purchased from Biovision Technology (Milpitas, CA, USA). Fetal bovine serum was purchased from Hangzhou Sijiqing Biological Engineering Material Co., Ltd., (Hangzhou, Zhejiang, China) and L-glutamine and MTT were purchased from Sigma (St Louis, MO, USA). Rabbit monoclonal anti-p53, -p62, -Beclin-1 and -light chain 3 (LC3) antibodies were purchased from Cell Signaling Technology (Beverly, MA, USA).

### Drug preparation

Rapamycin (Biovision Technology) was diluted in DMSO to create a stock solution that was stored according to the manufacturer’s instructions. The final concentrations of the rapamycin and CDDP solutions used were 5 and 2 μmol/l, respectively. This concentration of rapamycin was selected on the basis of our experiments on the MG63 cells.

### Cell culture and viability assay

The MG63 cells were maintained in RPMI-1640 medium containing 10% heat-inactivated fetal bovine serum and 0.03% L-glutamine, and incubated in a 5% CO_2_ atmosphere at 37ºC. The cells that were in a mid-log phase were used in the experiments. The cell viability was assessed using an MTT assay. To determine the time-course of the response of the MG63 cells to rapamycin and CDDP, the MG63 cells were plated into 96-well microplates (7×10^4^ cells/well). Rapamycin (5 μmol/l) and CDDP (2 μmol/l) was added to the culture medium and the cell viability was assessed using the MTT assay at 24, 48 and 72 h following the drug treatment. MTT solution (Sigma) was added to the culture medium (500 μg/ml final concentration) for 4 h prior to the end of treatment and the reaction was stopped by the addition of 100 μl 10% acidic SDS. The absorbance value (A) at 570 nm was read using an automatic multiwell spectrophotometer (Bio-Rad, Richmond, CA, USA). The percentage of cell deaths was calculated as follows: Cell death (%) = (1 − A of experimental well / A of positive control well) × 100.

### Detection of mitochondrial membrane potential (Δψ)

The mitochondrial Δψ was determined using the KeyGen Mitochondrial Membrane Sensor kit (KeyGen, Nanjing, Jiangsu, China). The mitosensor dye aggregates in the mitochondria of healthy cells and emits red fluorescence against a green monomeric cytoplasmic background staining. However, in cells with a collapsed mitochondrial Δψ, the dye is not able to accumulate in the mitochondria and remains as monomers throughout the cells emitting green fluorescence (19). Briefly, the MG63 cells were incubated with rapamycin and CDDP in 24-well plates for the indicated times and then pelleted, washed with PBS and resuspended in 0.5 ml diluted mitosensor reagent (1 μmol/ml in incubation buffer). Subsequent to incubating the cells with mitosensor reagent for 20 min, 0.2 ml incubation buffer was added and the cells were centrifuged and then resuspended in 40 μl incubation buffer. Finally, the cells were washed and resuspended in 1 ml PBS for flow cytometry analysis.

### Detection of the cell cycle

To analyze the effects of rapamycin on cell apoptosis progression, the MG63 cells were incubated with rapamycin and CDDP. The cells were harvested using 0.25% trypsin, washed with PBS, counted and adjusted to a concentration of 1×10^6^ cells/ml. The cells were fixed in 70% ethanol, treated with 100 mg/l RNase at 37ºC for 30 min and then stained with 50 mg/l propidium iodide (Sigma) for 30 min. The cells were analyzed using flow cytometry (Epics XL; Beckman Coulter, Fullerton, CA, USA).

### Total cell protein extraction and western blot analysis

For the extraction of the total cell proteins, the cells were washed with pre-cooled PBS and subsequently lysed in a pre-cooled RIPA lysis buffer containing 50 mM Tris-HCl (pH 7.4) 150 mM NaCl, 1 mM dithiothreitol (DTT), 0.25% sodium deoxycholate, 0.1% NP-40, 1 mM phenylmethysulfonyl fluoride (PMSF), 50 mM sodium pyrophosphate, 1 mM Na_3_VO_4_, 1 mM NaF, 5 mM EDTA, 5 mM EGTA and a protease inhibitor cocktail. Cell lysis was performed on ice for 30 min. Clear protein extracts were obtained by centrifugation at 12,000 × g for 30 min at 4ºC. The protein extraction procedure from the MG63 cells was performed as previously described. The protein concentration was determined using a Bradford protein assay kit (KeyGen). The proteins were resolved on 8.5% polyacrylamide gels and subsequently transferred onto nitrocellulose membranes. For immunoblotting, the nitrocellulose membranes were incubated overnight at 4ºC with specific antibodies recognizing the target proteins. The membranes were then incubated with a horseradish peroxidase (HRP)-conjugated secondary antibody (1:3,000) for 1 h at room temperature and subsequently analyzed using an enhanced chemiluminescence (ECL) detection system (Amersham Pharmacia Biotech*,* Amersham, UK) and visualized by autoradiograpy. β-actin proteins (1:5,000; Sigma) were used as loading controls.

### Statistical analysis

All data are presented as the mean ± SD. The statistical analysis was performed using an ANOVA followed by Dunnett’s t-test. P<0.05 was considered to indicate a statistically significant difference.

## Results

### Rapamycin inhibits cell viability and enhances the effects of CDDP-induced tumor cell growth inhibition

**Figure 1:**
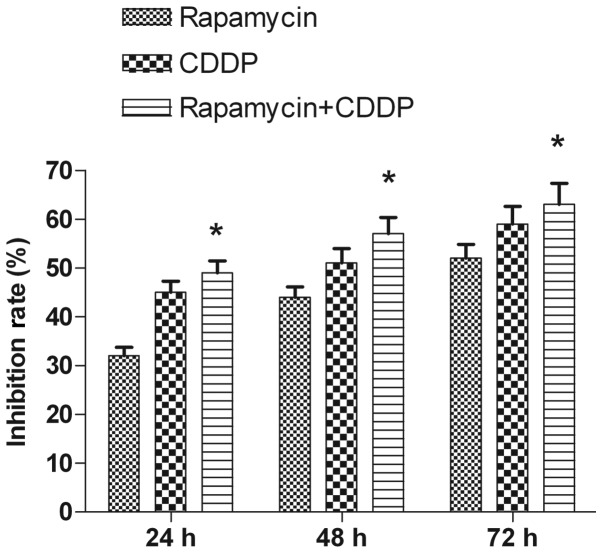
Viability of MG63 cells exposed to rapamycin or CDDP treatment. Reduced viability of MG63 cells was observed following rapamycin or CDDP treatment. The MG63 cells (7×10^4^ cells/ml) were cultured with rapamycin or CDDP for the indicated times and cell viability was analyzed using an MTT assay. Rapamycin had an increased effect when used in combination with CDDP compared with when used alone. Rapamycin inhibited the proliferation of the MG63 cells and enhanced the chemosensitivity of CDDP. Data are presented as mean ± SD of three independent experiments. ^*^P<0.05 vs. the rapamycin alone treatment group. CDDP, cis-diamminedichloroplatinum.

In the present study, rapamycin reduced MG63 cell viability in a time-dependent manner. The MTT assays revealed that following 24 h of treatment, the rate of inhibition reached 32±1.76% at the dose (5 μmol/l) used. The rate of inhibition increased when the incubation time was prolonged, reaching 44±2.09% at 48 h and 52±2.87% at 72 h following the treatment (Fig. 1). CDDP (2 μmol/l) was used to assess the clinical value of the mTOR inhibitor in the treatment of the tumor and to test the synergistic inhibitory effect of the mTOR inhibitor on the growth of the cells in combination with a chemotherapy drug. Rapamycin was shown to have an increased effect when used in combination with CDDP compared with when used alone (Fig. 1). Thus, rapamycin inhibited the proliferation of the MG63 cells and enhanced the chemosensitivity of CDDP.

### Rapamycin induces mitochondrial dysfunction and enhances the effects of CDDP-induced mitochondrial dysfunction

**Figure 2:**
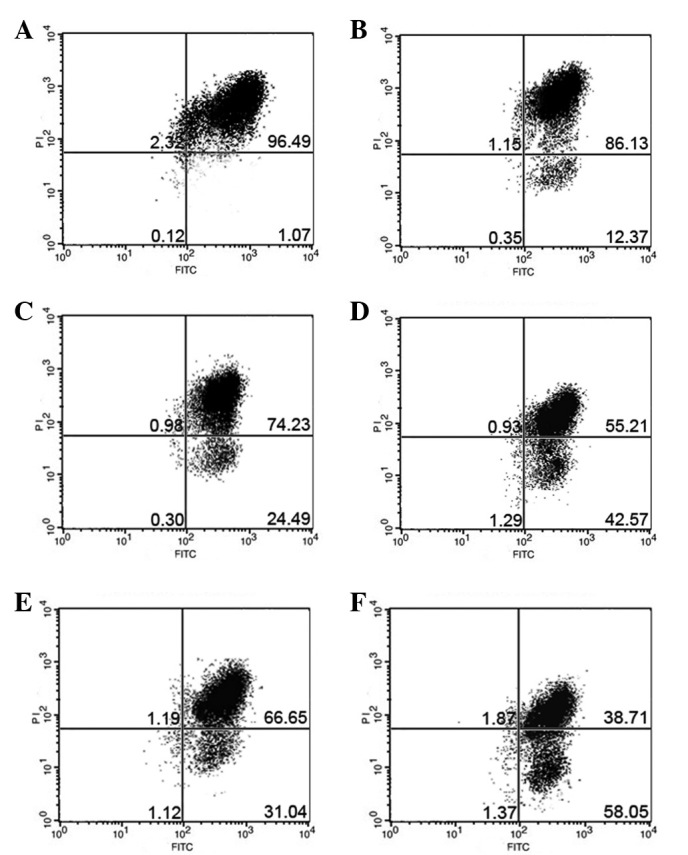
Mitochondrial dysfunction was induced following rapamycin or CDDP treatment. The MG63 cells were incubated with 5 μmol/l rapamycin or 2 μmol/l CDDP for the indicated times. (A) Control, (B) 6 h, (C) 12 h and (D) 24 h after rapamycin treatment (n=3). (E) 12 h with CDDP treatment. (F) 12 h with rapamycin and CDDP treatment. CDDP, cis-diamminedichloroplatinum.

In the present study, the mitochondrial Δψ was examined using the fluorescent dye, JC-1. A collapse in the mitochondrial Δψ was detected as early as 6 h after rapamycin or CDDP treatment, as indicated by an increased emission of green fluorescence. This change reached a maximum level following 24 h of rapamycin treatment or 12 h of CDDP treatment (Fig. 2). A collapse in mitochondrial Δψ indicates cell apoptosis or necrosis. Rapamycin used in combination with CDDP, rather than used alone, induced mitochondrial dysfunction and activated cell apoptosis in the MG63 cells. The present results demonstrate that rapamycin enhanced the effects of CDDP-induced mitochondrial dysfunction in the MG63 cells.

### Rapamycin induces apoptosis and enhances the effects of the CDDP-induced apoptosis of MG63 cells

**Figure 3:**
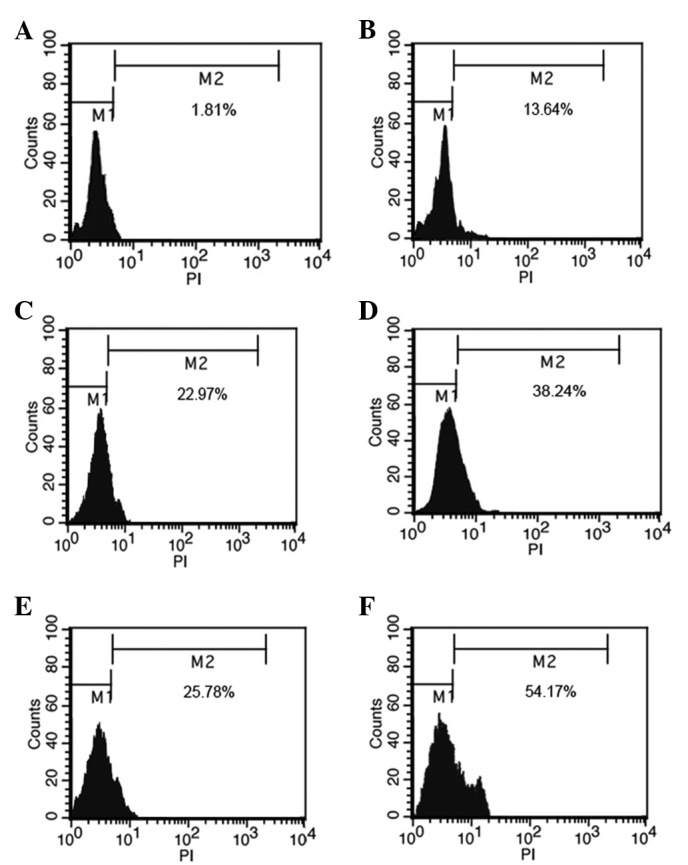
Apoptosis was induced following rapamycin or CDDP treatment. MG63 cells were incubated with 5 μmol/l rapamycin or 2 μmol/l CDDP for the indicated times. (A) Control, (B) 6 h, (C) 12 h and (D) 24 h following rapamycin treatment (n=3). (E) 12 h with CDDP treatment. (F) 12 h with rapamycin and CDDP treatment. CDDP, cis-diamminedichloroplatinum.

The effect of rapamycin on the cell apoptosis progression of the MG63 cells was studied following 6, 12 and 24 h of exposure to 5 μmol/l rapamycin. The flow cytometry analysis indicated that rapamycin induced cell apoptosis following 6, 12 and 24 h of treatment. There was a significant difference between the 5μmol/l rapamycin and CDDP groups and the control group at 12 h. When the cells were treated with rapamycin and CDDP at 12 h, the apoptosis of the MG63 cells was significantly increased than when rapamycin was used alone (Fig. 3). The results indicated that rapamycin enhanced the effects of inducing MG63 cell apoptosis by CDDP.

### Rapamycin increases the expression of p62 and LC3 and enhances the effects of CDDP-activated autophagy

**Figure 4:**
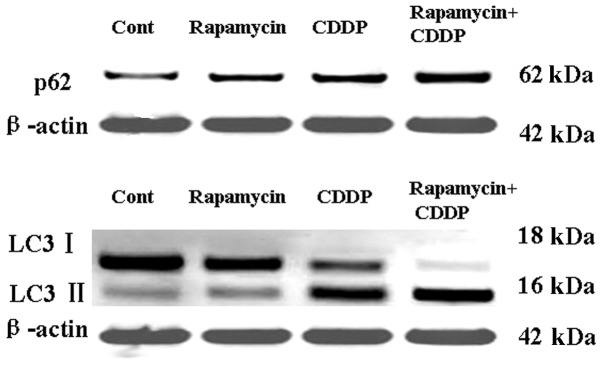
Western blotting analysis of p62 and LC3 of the control and rapamycin- or CDDP-treated MG63 cells. The cells were treated with 5 μmol/l rapamycin or 2 μmol/l CDDP for 12 h and harvested for the total proteins. The results indicate that rapamycin may upregulate the expression of p62 and LC3II. Rapamycin enhanced the effects of CDDP in the upregulation of p62 and LC3II expression. CDDP, cis-diaminodichloroplatinum; LC3, light chain 3.

To distinguish the specific inhibition of mTOR-mediated cell proliferation from autophagy, the expression of the autophagic proteins, LC3 and p62, was measured following treatment with rapamycin or CDDP. As shown in Fig. 4A, the expression of p62 in the MG63 cell line was activated by rapamycin or CDDP treatment, and rapamycin may have enhanced the effects of CDDP-activated autophagy. As shown in Fig. 4B, western blotting analysis was used to detect the protein levels of LC3-I and LC3-II. The results revealed that the levels of LC3, particularly LC3-II, had increased, leading to an increased ratio of LC3-II/LC3-I following rapamycin or CDDP treatment. Rapamycin may have thus enhanced the effects of CDDP-activated autophagy. These results suggest that rapamycin may induce tumor cell apoptosis by regulating p62 and LC3II.

### Rapamycin upregulates p53 and Beclin-1 and enhances the effects of CDDP in the upregulation of p53 and Beclin-1

**Figure 5:**
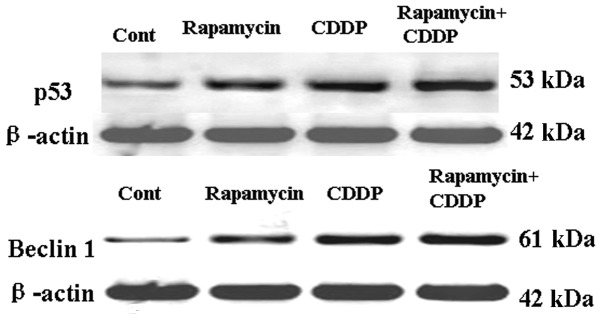
Western blotting analysis of Beclin-1 and p53 of the control and rapamycin- or CDDP-treated MG63 cells. The cells were treated with 5 μmol/l rapamycin or 2 μmol/l CDDP for 12 h and harvested for the total proteins. The results indicate that rapamycin may upregulate the expression of Beclin-1 and p53. Rapamycin enhanced the effects of CDDP in the upregulation of Beclin-1 and p53 expression. CDDP, cis-diaminodichloroplatinum.

To distinguish the specific inhibition of mTOR-mediated cell proliferation from autophagy and apoptosis, the expression of the autophagic and apoptotic proteins, p53 and Beclin-1, was measured following treatment with rapamycin or CDDP. As shown in Fig. 5A, the expression of Beclin-1 in the MG63 cell line was activated by rapamycin or CDDP treatment and rapamycin may have enhanced the effects of CDDP-induced autophagy and apoptosis. As shown in Fig. 5B, the expression of p53 in the MG63 cell line was also upregulated and rapamycin may have enhanced the effects of CDDP-induced apoptosis. These results suggest that rapamycin may induce tumor cell apoptosis by regulating Beclin-1 and p53.

## Discussion

The tumor suppressor, p53, plays a central role in sensing various genotoxic stresses. p53 is known to play significant roles in apoptosis by regulating the expression of proapoptotic proteins (20,21). The present study demonstrated that as an inhibitor of autophagy, rapamycin significantly upregulated the levels of p53, indicating that apoptosis may be triggered by CDDP. The mitochondrial Δψ was shown to collapse following rapamycin treatment. The study revealed that rapamycin induced mitochondrial dysfunction in the MG63 cells. Mitochondria play a central role in regulating cell death and survival. Diverse proapoptotic stimuli act on mitochondria, triggering mitochondrial Δψ collapse, cytochrome c release and caspase activation. The mitochondrial permeability transition (MPT) represents a significant event in initiating apoptotic cell death (22).

Increasing evidence suggests that autophagy plays significant roles in tumor cell growth, differentiation and the response to anti-tumor drugs (23). Numerous classical anti-tumor drugs have now been identified to exert their cytotoxic actions by autophagic mechanisms (24–26). In the present study, the inhibition of mTOR by rapamycin resulted in a significant increase in the levels of p62, LC3 and Beclin-1, and a particularly increased production of LC3-II. LC3 is an autophagosomal ortholog of yeast Atg8. LC3 has been best characterized as an autophagosomal marker in mammalian autophagy and the levels of LC3 may also reflect the levels of autophagy (27). Beclin-1 is the mammalian ortholog of the yeast ATG6-Vps30 gene. p62 is also present in protein aggregates that are positive for ubiquitin and microtubule-associated protein-1 (MAP1)LC3, a well-characterized marker of a cellular process known as autophagy. p62 is believed to be the link between polyubiquitinated proteins and autophagy (28). The present results suggest that autophagy inhibited by rapamycin may contribute to cell apoptosis.

In summary, the present study revealed a new mechanism associated with mTOR inhibition that triggered the impairment of cell proliferation and the induction of the cell death of cancer cells. The inhibition of mTOR increases the expression of p53 and induces the expression of the proapoptotic and autophagic proteins, p62, LC3 and Beclin-1. Rapamycin induced the death of cancer cells through apoptotic and autophagic mechanisms and may have enhanced the effects of CDDP on activating autophagy and inducing apoptosis. Further investigation of the association between autophagy activation and the anti-tumor effects of mTOR inhibitors may unveil new strategies for tumor therapy.
